# Enhancement of Stress Granule Formation by a Chiral Compound Targeting G3BP1 via eIF2α Phosphorylation

**DOI:** 10.3390/ijms251910571

**Published:** 2024-09-30

**Authors:** Yoon Ho Park, Hyun Suh Cho, Sungjin Moon, Sim Namkoong, Hyun Suk Jung

**Affiliations:** 1 Department of Biochemistry, Kangwon National University, Chuncheon 24341, Republic of Korea; yhpark99@kangwon.ac.kr (Y.H.P.); kevincho96@kangwon.ac.kr (H.S.C.); 2 Department of Biological Science, Kangwon National University, Chuncheon 24341, Republic of Korea; sungjin.moon@kangwon.ac.kr

**Keywords:** chiral compound, stress granule, drug discovery, G3BP1, eIF2α phosphorylation

## Abstract

The chirality of a chemical differentiates it from its mirror-image counterpart. This unique property has significant implications in chemistry, biology, and drug discovery, where chiral chemicals display high selectivity and activity in achieving target specificity and reducing attrition rates in drug development. Stress granules (SGs) are dynamic assemblies of proteins and RNA that form in the cytoplasm of cells under stress conditions. Modulating their formation or disassembly could offer a novel approach to treating a wide range of diseases. This has led to significant interest in SGs as potential therapeutic targets. This study examined the NTF2-like domain of G3BP1 as a possible target for SG modulation. Molecular docking was used to simulate the interactions of compounds with the domain, and a potential candidate with a chiral structure was identified. The experiments showed that the compound induced the formation of SG-like granules. Importantly, the ability of this compound to modulate SG offers valuable insights into a new mechanism underlying the dynamics and promoting the assembly of SGs, and this new mechanism, in turn, holds potential for the development of drugs with diverse mechanisms of action and potentially synergistic effects.

## 1. Introduction

Stress granules (SGs) are dynamic, non-membranous aggregates of proteins and RNAs that form in the cytoplasm when cells are exposed to various stressors, such as heat shock, oxidative stress, or viral infection [1]. SGs play a crucial role in regulating mRNA translation and cell survival under stress conditions by sequestering mRNAs and translation initiation factors, thereby repressing translation [2]. Moreover, SGs have been implicated in various diseases, including neurodegenerative disorders (such as Alzheimer’s, Parkinson’s, and Amyotrophic Lateral Sclerosis), cancer, and viral infections (e.g., SARS-CoV-2 and Coxsackievirus B3) [1,3,4,5,6]. Therefore, understanding the molecular mechanisms underlying SG formation and regulation is of great importance for developing novel therapeutic strategies.

G3BP1 (Ras GTPase-activating protein-binding protein 1) is a multifunctional RNA-binding protein that plays a central role in SG assembly [7,8]. G3BP1 contains several domains, including an N-terminal NTF2-like (NTF2L) domain, an acid-rich domain, and an RRM domain [8]. The NTF2L domain of G3BP1 is essential for its dimerization and SG nucleation [8]. Interestingly, this domain also interacts with various proteins containing FGDF motifs, such as USP10 and viral proteins, which can modulate SG formation [9,10,11]. Stress-granule formation is known to be regulated through several pathways, including the well-characterized eIF2α phosphorylation pathway, the mTOR-S6K pathway, and the eIF4A inhibition pathway [12,13,14]. While these pathways have been extensively studied, the potential of directly targeting the NTF2L domain of G3BP1 with small molecules remains largely unexplored. Apart from our previous research, there have been no specifically targeted inhibitors discovered for this domain. Therefore, the identification of diverse small molecules targeting the NTF2L domain and the analysis of their interactions are both crucial for expanding our understanding of stress granule-regulation mechanisms. This approach could not only provide new insights into SG dynamics but also pave the way for developing potential therapeutic interventions for SG-related diseases.

Chiral compounds are molecules that exist in two non-superimposable mirror-image forms that are known as enantiomers. Enantiomers can exhibit distinct biological activities due to their differential interactions with chiral targets, such as proteins [15]. In the context of SG regulation, we hypothesize that chiral compounds targeting the NTF2L domain of G3BP1 could modulate SG formation by differentially influencing the protein–protein interactions mediated by this domain. Specifically, one enantiomer of a chiral compound might stabilize the dimeric state of G3BP1 and promote SG assembly, whereas the other enantiomer could disrupt G3BP1 dimerization and inhibit SG formation. This stereospecific modulation of G3BP1 function could provide a novel and precise approach to control SG dynamics and potentially treat SG-related diseases.

In this study, we aimed to identify chiral compounds that target the NTF2L domain of G3BP1 using in silico molecular-docking simulations. We then investigated the effects of the identified compounds on SG formation in cell-based assays. Our findings provide new insights into the role of chirality in modulating SG dynamics and highlight the potential of chiral compounds as therapeutic agents for SG-related disorders.

## 2. Results

### 2.1. Identifying Promising Chiral Compounds for Binding to the NTF2-like Domain of G3BP1

**Figure 1:**
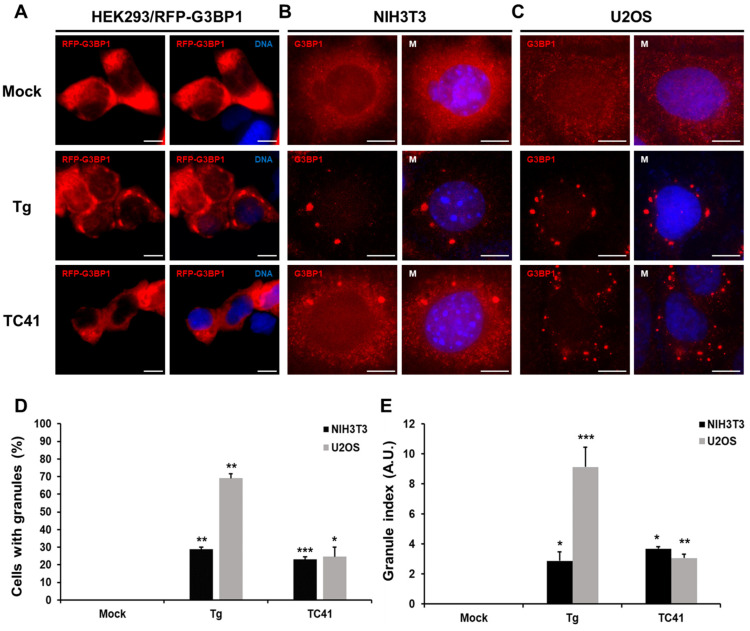
SG-like G3BP1-positive granules induced by TC41, the top-ranked compound with high chirality and high affinity. (**A**) HEK293 cells continuously expressing RFP-G3BP1, (**B**) NIH3T3 cells and (**C**) U2OS cells were treated with DMSO (1:1000), 1 µM of thapsigargin (Tg) and 5 µM of the top-ranked compound 41 (TC41) for 2 h. G3BP1-positive granules were visualized either through RFP-G3BP1 in HEK293 cells or via G3BP1 immunofluorescence staining. Scale bars, 10 μm. (**D**) Percentage of NIH3T3 and U2OS cells producing granules was determined based on G3BP1 immunofluorescence (mean ± SEM; n ≥ 3, * *p* < 0.05; ** *p* < 0.01; *** *p* < 0.001 in Student’s *t*-test). (**E**) Quantification of G3BP1 positive granules in U2OS cells using the Granule index (granule area/cell area), based on G3BP1 immunofluorescence. Data are shown in arbitrary units (a.u.) (mean ± SEM; n ≥ 3, * *p* < 0.05; ** *p* < 0.01; *** *p* < 0.001 in Student’s *t*-test).

G3BP1 is known as the key protein for SG formation, characterized by its essential NTF2L domain. This domain is integral to the processes of nuclear localization, interactions among proteins, and binding with RNA [8]. The FGDF motifs that were confirmed to engage with the NTF2L domain of G3BP1/2 in previous studies were shown capable of SG formation [16,17]. To discover the potential drug candidate, a molecular-docking simulation was performed. Based on the virtual screening result, we analyzed the top-ranked compounds (TCs) and selected the chiral compound with the highest affinity (−8.6 kcal/mol). Subsequently, we investigated whether TC41, the selected compound, could stimulate the formation of SG-like G3BP1-positive granules in cells by treating HEK293 cells expressing RFP-G3BP1, NIH3T3, and U2OS cells with 5 µM of each compound for 2 h (Figure 1A–C). To establish a benchmark for SG formation, cells were exposed to thapsigargin (Tg, 1 µM), a known ER stress inducer, serving as a positive control. DMSO was utilized as the negative control. Importantly, the administration of TC41 resulted in the appearance of distinct red foci within the cells, observed in both RFP-G3BP1 and endogenous G3BP1. This finding demonstrates TC41’s capacity to induce the formation of SG-like G3BP1-positive granules in the cytoplasm. Two separate measurement criteria were used to more quantitatively assess granule formation. The first criterion, referred to as “Cells with granules (%)”, represents the proportion of cells containing G3BP-positive granules in comparison to the total cell count observed. The second criterion, referred to as the “Granule Index”, quantifies the prevalence of SG by calculating the area of the granules relative to the total visualized cellular field. Combined, these two measurement criteria provide a thorough quantification of the efficacy and extent of granule induction by the compounds. By using these approaches, we analyzed the formation of G3BP1-positive granules in NIH3T3 and U2OS cells in response to TC41 or Tg, respectively (Figure 1D,E). Upon Tg treatment, 70% of U2OS cells displayed SG formation, in contrast to only 30% of NIH3T3 cells. However, treatment with TC41 resulted in similar induction rates in both cell types, with 20% of cells exhibiting SG formation (Figure 1D). In U2OS cells, the Granule index revealed a notable Tg score of 9.11, in contrast to TC41’s diminished score of 3.05. However, in the NIH3T3 cells, Tg exhibited a pronounced score of 2.86, whereas TC41 presented a higher score of 3.68 (Figure 1E). These data show that while TC41 has the ability to induce granule formation, it generates fewer granules than Tg.

### 2.2. Dependence of Induced Formation of G3BP1-Positive Granules by TC41 on eIF2α Phosphorylation without Exhibiting Toxicity

**Figure 2:**
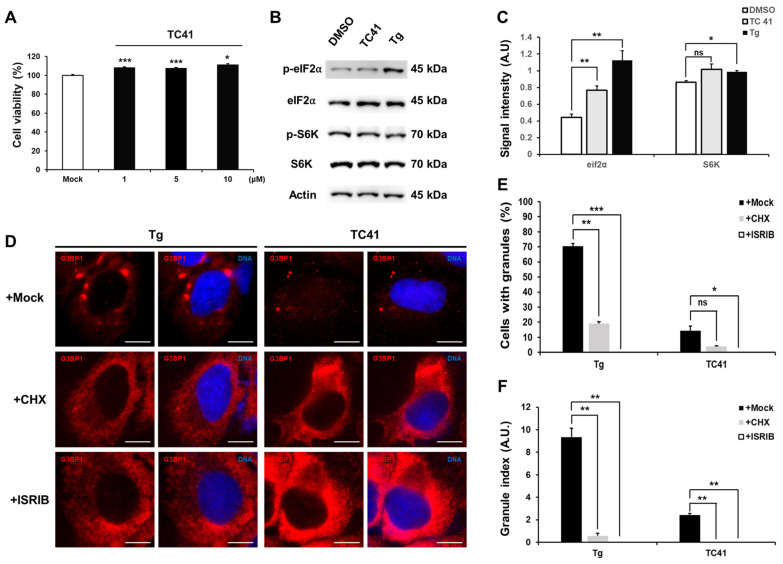
Dependence of induced formation of SG-like G3BP1-positive granules by TC41 on eIF2⍺ phosphorylation. (**A**) U2OS cells were exposed to increasing concentrations of TC41 (1,5,10 µM) for 24 h. Cell viability was determined using MTT assay (mean ± SEM; *n* = 3, * *p* < 0.05; *** *p* < 0.001 in Student’s *t*-test). (**B**) U2OS cells treated with 5 µM of TC41 for 2 h. Immunoblotting of the whole cell lysate was used to assess the phosphorylation statuses of eIF2a and S6K, using specific antibodies. Actin was a loading control for the assay. (**C**) Relative intensity of p-S6K, p-eIf2α in each condition (mean ± SEM; *n* = 3, * *p* < 0.05; ** *p* < 0.01, ns, non-significant in Student’s *t*-test). (**D**) U2OS cells exposed to either Tg (1 µM) or TC41 (5 µM) either alone or in combination with cycloheximide (CHX, at 2 μg/mL) or ISRIB (a p-eIF2⍺ inhibitor, at 200 nM) for 2 h. G3BP1-positive granules were visualized through immunofluorescence staining (Scale bars: 10 µm). (**E**) The proportion of U2OS cells forming granules was calculated based on G3BP1 immunofluorescence (mean ± SEM; n ≥ 3, * *p* < 0.05; ** *p* < 0.01; *** *p* < 0.001, ns, non-significant, Student’s *t*-test). (**F**) G3BP1 positive granules in U2OS cells were quantified using the Granule index (granule area/cell area), derived from G3BP1 immunofluorescence. Results are presented in arbitrary units (a.u.) (mean ± SEM; n ≥ 3, ** *p* < 0.01; in Student’s *t*-test).

We evaluated the potential cytotoxicity of TC41 on U2OS cells by treating them with various concentrations of this compound (0, 1, 5 and 10 µM) for 24 h, followed by an MTT assay (Figure 2A). TC41 did not have any cytotoxic effects on U2OS cells.

The formation of SG is often related with the phosphorylation of the translation initiation factor eIF2α [7,18]. We used Western blotting to investigate whether the G3BP1-positive granules induced by TC41 are generated through an eIF2α-dependent pathway (Figure 2B,C). Our data demonstrated, as was previously known, that the ER stress inducer Tg phosphorylates eIF2α [19]. However, TC41 induced only minimal phosphorylation of eIF2α. Since the mTOR-S6 kinase is known to facilitate SG formation [7], we also compared its activity. While Tg significantly upregulated mTOR-S6 kinase activity compared to the control, TC41 did not show a substantial difference. These results indicate that TC41 induces the formation of G3BP1-positve granules through eIF2α phosphorylation rather than the S6K pathway.

Additional confirmation of the dependence of the induced formation of G3BP1-positive granules on eIF2α phosphorylation was obtained by testing for granule formation with ISRIB, known as a p-eIF2α signaling inhibitor. Additionally, to ascertain that TC41 induces G3BP1-positive granule formation through an alternative pathway, we assessed granule formation using cycloheximide (CHX), known as an SG suppressor (Figure 2D). CHX inhibits SG assembly by blocking protein synthesis. It does this by preventing untranslated mRNA accumulation and disrupting the formation of translational initiation complexes [20]. Our results showed that Tg demonstrated effective inhibition of granule formation in the presence of both CHX and ISRIB. However, in the case of TC41, although CHX reduced the granule formation capability to a certain extent, ISRIB effectively suppressed this formation. The Granule index exhibited a similar pattern to the granule formation capability (Figure 2E,F). Considering the collective results, our data indicate that the G3BP1-positive granules induced by TC41 closely resemble the stress granules formed by Tg.

### 2.3. Chirality Analysis of TC41

**Figure 3:**
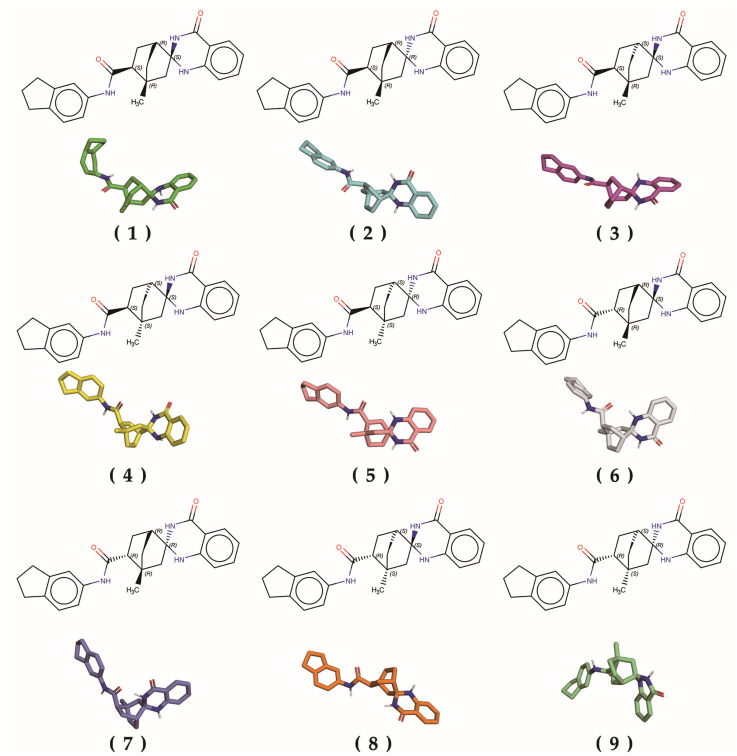
Two- and three-dimensional conformations of stereoisomers of TC41 targeting the G3BP1 NTF2-like domain. The stereoisomers are labeled (**1**) to (**9**), and their corresponding 2D chemical structures and 3D conformations are presented. The notations Rectus (R) and Sinister (S) in the 2D structures indicate the absolute configuration of the chiral centers.

The chemical structure of the compound was easily confirmed to be chiral as a result of the presence of an asymmetric carbon atom. To further analyze the detailed chirality of the TC41, we employed a combination of computational methods, including the generation of stereoisomers using the stereochemistry flags calculators of Achieving Chemistry Excellence: Organic Chemistry and Marvin software (version 23.17), followed by the conversion of 2D structures to 3D using Open Babel (Figure 3).

### 2.4. Molecular-Docking Simulations of Stereoisomers with G3BP1

**Figure 4:**
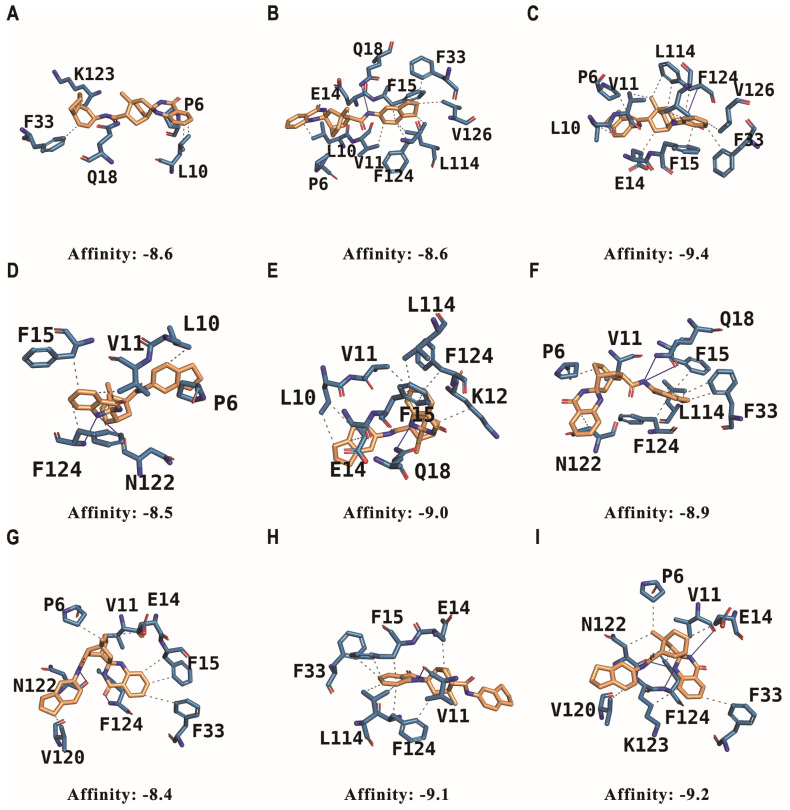
Best docking poses of the stereoisomers in the complex with the G3BP1 NTF2-like domain. In the figure, the stereoisomers are represented by brown sticks, whereas the G3BP1 protein is depicted as blue sticks. The key residues involved in the interactions between the stereoisomers and G3BP1 are labeled as one letter of the amino acid code. Hydrogen bonds are indicated by blue lines, and hydrophobic interactions are presented as grey dotted lines. The binding affinities (kcal/mol) for each stereoisomer are provided below the respective panels. Respectively, (**A**–**I**) represent the interactions of stereoisomers (**1**–**9**) with the G3BP1 NTF2-like domain.

The molecular-docking simulations of the nine stereoisomers with G3BP1 revealed distinct binding characteristics for each isomer, as described in Table 1. The binding affinities of the stereoisomers ranged from −8.4 to −9.4 kcal/mol, thereby indicating strong interactions with the G3BP1 receptor. Stereoisomer (**3**) exhibited the highest affinity (−9.4 kcal/mol), followed by stereoisomers (**9**) and (**8**), with affinities of −9.2 and −9.1 kcal/mol, respectively (Figure 4).

### 2.5. Analysis of the Interaction of Stereoisomers with G3BP1 in Comparison to the FGDF Motif

Previous studies have elucidated the crystal structure of the G3BP2 NTF2L in complex with a canonical FGDF motif peptide (PDB ID: 5DRV). These studies revealed the key residues involved in the interaction between the FGDF peptide and G3BP2. Moreover, the NTF2L domains of both G3BP1 and G3BP2 have been shown to exhibit high sequence homology and share identical interaction residues. The key interactions between the FGDF peptide and G3BP2 involve the F3 and F6 residues of the FGDF motif, which are accommodated in hydrophobic pockets formed by the G3BP2 residues V11, F15, F33, L22, and R32. The L1 residue of the FGDF peptide is located in another hydrophobic pocket composed of L10, V11, and F124. Additionally, the backbone of the FGDF peptide forms hydrogen bonds with the G3BP2 residues N122 and F124.

A comparison of the docking results of the stereoisomers with these canonical interactions led to the discovery that the stereoisomers largely maintain the key hydrophobic interactions involving F3 and F6. However, the docking results also highlight additional significant interactions. P6, L10, and V11 of G3BP1 were found to engage in hydrophobic interactions with eight out of the nine stereoisomers. This suggests that these residues play a crucial role in stabilizing the binding of the chiral compound beyond the interactions observed in the FGDF motif binding mode.

Apart from the above-mentioned interactions, the stereoisomers exhibit distinct hydrogen-bonding patterns with G3BP1. Although all the stereoisomers form hydrogen bonds with N122 and F124 by mimicking the backbone interactions of the FGDF peptide, specific stereoisomers undergo additional hydrogen-bonding interactions. For instance, stereoisomer (**3**), which has the highest binding affinity, forms a hydrogen bond with F124, similar to the FGDF peptide. In contrast, stereoisomers (**1**), (**2**) and (**6**) form hydrogen bonds with Q18, while stereoisomers (**4**), (**5**) and (**7**) interact with N122 and F124 through hydrogen bonding.

These specific hydrogen-bonding patterns might contribute to the differences in the binding affinities observed among the stereoisomers. The additional hydrogen bonds formed by the high-affinity stereoisomers, such as (**3**), may enhance the stability of the complex and lead to improved binding affinity. Conversely, the lower affinity stereoisomers may lack these additional stabilizing interactions or may exhibit suboptimal hydrogen-bonding patterns.

## 3. Discussion

The cell-based assays demonstrated that TC41, a chiral compound identified through molecular-docking simulations, effectively induced the formation of SG-like G3BP1-positive granules in HEK293, NIH3T3, and U2OS cells. This indicates that TC41 can promote the assembly of SGs, a key cellular process in stress response and disease pathogenesis. Interestingly, even though the pattern of granule formation induced by TC41 resembled that of the known SG inducer thapsigargin (Tg), it produced fewer granules, suggesting potential differences in their mechanisms of action.

Importantly, the ability of TC41 to induce the formation of SGs without exhibiting cytotoxicity highlights its potential as a safe and effective modulator of SG dynamics. A more in-depth investigation into the molecular mechanism of TC41-induced SG formation revealed that it was dependent on eIF2α phosphorylation but independent of the mTOR-S6K pathway. In contrast, the finding that Tg activated both pathways indicates that SG induction by TC41 and Tg proceeds via distinct mechanisms.

The dependence of TC41-induced SG formation on eIF2α phosphorylation was further confirmed by the inhibitory effect of ISRIB, an inhibitor of eIF2α phosphorylation. Conversely, cycloheximide (CHX), a known inhibitor of SG formation, only partially limits the induced formation of granules by TC41. These findings suggest that TC41 promotes SG assembly primarily through the eIF2α-dependent pathway, thereby providing new insights into the regulation of SG dynamics.

The interaction shown to occur between TC41 and the G3BP1 demonstrated that the stereochemistry of the chiral compound significantly influences its binding pose and affinity to G3BP1. The different binding energies calculated for the nine stereoisomers ranged from −8.4 to −9.4 kcal/mol and are indicative of differential binding strengths. Stereoisomer (**3**) had the highest affinity (−9.4 kcal/mol), whereas stereoisomers (**7**) and (**4**) had lower affinities of −8.4 and −8.5 kcal/mol, respectively. Our analysis of the binding poses revealed that the stereoisomers engage in key hydrophobic interactions with G3BP1 residues, similar to the canonical FGDF motif. However, additional hydrophobic interactions involving P6, L10 and V11 were observed in most stereoisomers, suggesting their important role in stabilizing the binding. Furthermore, distinct hydrogen-bonding patterns were identified among the stereoisomers, with high-affinity stereoisomers forming additional hydrogen bonds that may contribute to enhanced binding affinity.

Further studies would be necessary to elucidate the detailed molecular interactions between TC41 and G3BP1, as well as to characterize the downstream effects of TC41-induced SG formation on cellular functions and disease pathogenesis. Additionally, the structure–activity relationship of TC41 and its analogs would have to be investigated to optimize their drug-like properties and explore their therapeutic potential in SG-related disorders.

In conclusion, this study aimed to elucidate novel mechanisms regulating stress granule (SG) dynamics and explore potential therapeutic approaches for SG-related disorders. We identified TC41 as a novel chiral compound capable of inducing SG formation through the eIF2α pathway by targeting the NTF2L domain of G3BP1. The stereochemistry-dependent binding and biological activity of TC41 provide new insights into the regulation of SG dynamics and highlight the potential of chiral compounds as modulators of SG-related processes. These findings not only advance our understanding of SG biology but also provide a framework for the development of novel SG-targeted therapeutics. Our work paves the way for future investigations into the role of SG in health and disease, potentially leading to innovative treatment strategies for conditions involving SG dysregulation.

## 4. Material and Methods

### 4.1. In Silico Molecular-Docking Simulation and Chiral Structure Analysis

To identify potential hit compounds capable of acting as G3BP1 agonists, molecular-docking simulations were performed utilizing the AUTODOCK VINA software [21]. The docking simulation employed the protein structure of G3BP1 (PDB ID: 4FCJ) as the receptor [22]. The docking search space was set to include the FGDF motif binding site in the NTF2L Domain of G3BP1, which is known to be crucial for protein–protein interactions and SG formation [16]. A compound library containing 578,124 compounds was obtained from the Korea Chemical Bank of Korea Research Institute of Chemical Technology www.chembank.org (accessed on 13 February 2023). The docking results were ranked based on affinity, with a cutoff of −8.0 kcal/mol. The top 72 compounds meeting this criterion were obtained from the Korea Chemical Bank for further analysis.

These top-ranked compounds were then tested using a cell-based assay and selected based on their effectiveness and chirality. The chiral structures obtained through chiral structure analysis were subjected to the same docking simulation settings to further investigate their binding properties.

The chirality of the compound was calculated using the stereochemistry flag calculators of Achieving Chemistry Excellence: Organic Chemistry https://epoch.uky.edu/ace/ (accessed on 27 January 2024), and this result was used to specify parameter values for generating the structures of the chiral compounds by Marvin 23.17.0, Chemaxon https://www.chemaxon.com (accessed on 29 January 2024). The parameter values were set to tetrahedral stereo, and the maximum number of stereoisomers was specified to be 16. Each of the 2D stereoisomers was converted to the corresponding 3D structure by Open Babel software [23]. Then, docking simulation was performed by using the structures of these 3D stereoisomers as ligands and the G3BP1 structure as a receptor. Interactions were analyzed using the Protein–Ligand Interaction Profiler (PLIP) tool to identify the interactions between these stereoisomers and G3BP1 [24]. Instrumentation was used in Kangwon center for systems imaging.

### 4.2. Formation of a Reliable Cell Line That Demonstrates Expression of mEmerald-LaminB1 and RFP-G3BP

The pmEmerald-LaminB1-10 vector was obtained from the Addgene plasmid repository (#54140, deposited by Dr. Michael Davison) [18]. We obtained the pRFP-G3BP plasmid through the generosity of Dr. B.L. Wolozin (Boston University) and Dr. N. Kedersha (Harvard) [25]. For cell transfection, we used polyethylenimine (Polysciences, Warrington, PA, USA, catalog number 23966) to introduce both plasmids into HEK293 cells. Following transfection, we applied puromycin selection (15 µg/mL, Sigma-Aldrich, St. Louis, MO, USA, P8833) for a period of 3 days. Subsequently, we performed single-cell seeding, distributing individual cells into separate wells. To confirm successful expression of mEmerald-Laminb1 and RFP-G3BP, we utilized fluorescence microscopy for visualization and analysis.

### 4.3. Cell Culture

HEK293 cells that continuously express mEmerald-Laminb1 and RFP-G3BP and NIH3T3 cells were cultured in Dulbecco’s Modified Eagle Medium (DMEM) (WELGENE, Gyeongsan-si, Republic of Korea, LM 001-05) with 10% fetal Bovine Serum (FBS) (Gibco, Waltham, MA, USA, 16000069) and 1% Antibiotic–Antimycotic Solution (A/A) solution (WELGENE, LS 203-01) at 37 °C, under 5% CO_2_. U2OS cells were cultured in Roswell Park Memorial Institute 1640 (RPMI-1640) (WELGENE, LM 011-01) with 10% FBS and 1% A/A solution at 37 °C in 5% CO_2_.

### 4.4. Compound Treatments

The cells were seeded and treated with 5 µM of the G3BP1-targeted top-ranked compound (TC41) for 2 h (TC41; 5 mM stock diluted with DMSO). Cells were treated with 1 µM of thapsigargin (Tg; Sigma, Kanagawa, Japan, T9033), known as an ER stress inducer, as the positive control, and dimethyl sulfoxide (DMSO; Sigma, D8418) diluted at 1:1000 was used as the negative control. To identify the ability of TC41 to induce the formation of SG, 2 μg/mL of cycloheximide (CHX; Sigma, C7698) was co-treated with either TC41 or Tg. To evaluate the dependence of SG formation on eif2α kinase, 200 nM of the Integrated Stress Response Inhibitor (ISRIB; Selleckchem, Houston, TX, USA, S7400) was co-treated with TC41 or Tg.

### 4.5. Immunofluorescence Imaging and Quantification of Granule Formation

HEK 293 stable cells, NIH3T3 cells, and U2OS cells were seeded on the coverslips in a 24-well plate (Cat. 32024, SPL Life Science, Pocheon-si, Republic of Korea). After compound treatment, cells were fixed with 4% paraformaldehyde (PFA) (EMS, Osaka, Japan, 15710) for 20 min at room temperature, permeabilized with 0.2% Triton X-100 (Sigma, T9284) for 30 min in an ice bucket, and blocked with 3% bovine serum albumin (BOVOGEN, East Keilor, VIC, Australia, BSAS-0.1) for 1 h at room temperature. The 4% PFA, 0.2% Triton X-100, and 3% BSA were diluted in PBS. The cells were incubated with the primary antibody, the G3BP1 antibody (1:1000; Santa Cruz Biotechnology, Dallas, TX, USA, sc-365338), for 2 h at room temperature. This was followed by incubation with the secondary antibody, Alexa fluor-conjugated secondary antibodies (1:2000; Invitrogen, Waltham, MA, USA, anti-mouse Cat. A32744), at room temperature for 1 h. The cells were washed with PBS, counterstained with DAPI, and mounted in anti-fade mounting media (H-1000, Vector Laboratories, Newark, CA, USA). Images were captured with Thunder Imager (Leica, Wetzlar, Germany). Granules were estimated with ImageJ https://imagej.nih.gov/ij (accessed on 30 October 2020), using a previously described method [26]. The Granule Index was calculated by comparing the overall area of the stress granule to the cell area in each field and then finding the average across all fields. The “Cells with granules (%)” index was calculated by counting the number of cells containing stress granules in each image. This number was then divided by the total number of cells in that field to calculate the percentage.

### 4.6. Assessment of Cell Viability

U2OS cells were cultured in 24-well plates and subjected to various concentrations of TC41 for a 24-h duration. We assessed cell viability using the MTT ([4,5-dimethylthiazol-2-yl]-2,5-diphenyltetrazolium bromide) assay. This procedure was conducted in accordance with our previously described methodology [27].

### 4.7. Immunoblotting

U2OS cells were lysed with cell lysis buffer (Tris pH 7.5, 150 mM NaCl, 1% NP-40, 1 mM NaF, 0.1 mM NaVO_3_, 1× EDTA-free protease inhibitor cocktail) (Roche, 05 892 791 001, Basel, Switzerland). The protein concentration of each sample was normalized by the Bradford assay, after which lysis was completed, and the protein samples were boiled in 6X SDS buffer at 95 °C for 5 min. Protein separation was performed using 10% SDS-PAGE, followed by transfer onto either PVDF or nitrocellulose membranes. The membranes underwent a blocking step prior to antibody incubation. We utilized primary antibodies for the following proteins: phospho-eIF2α (Cell Signaling, Danvers, MA, USA, 9721S), eIF2α (sc-133132), phospho-S6K (sc-8416), and S6K (sc-8418), each diluted at 1:1000. Additionally, an actin antibody (sc-8432) was used at a 1:2000 dilution. With the exception of phospho-eIF2α, all antibodies were sourced from Santa Cruz Biotechnology, at 4 °C for 16 h. Finally, the samples were probed with horseradish peroxidase-conjugated secondary antibodies (1:2000 for anti-rabbit (1706515) and anti-mouse (1706516)). Chemiluminescence images were acquired using an ImageQuant^TM^ 500 imaging system (Amersham, Amersham, UK).

## Figures and Tables

**Table 1:** Binding affinities and interaction analysis of the stereoisomers with the G3BP1 NTF2-like domain.

Conformation Number	Affinity (kcal/mol)	Hydrophobic Interaction	Hydrogen Interaction
(1)	−8.6	P9, L10, F33, K123	Q18
(2)	−8.6	P6, L10, V11, E14, F15, F33, L114, F124, V126	Q18
(3)	−9.4	P6, L10, V11, E14, F15, F33, L114, F124, V126	F124
(4)	−8.5	P6, L10, V11, F15, F124	N122, F124
(5)	−9.0	L10, V11, E14, F15 L114, K123, F124	Q18
(6)	−8.9	P6, V11, F15, F33, L114, N122, F124	Q18
(7)	−8.4	P6, V11, E14, F15, F33, V120	N122, F124
(8)	−9.1	E14, F15, F33, L114, F124	V11
(9)	−9.2	P6, E14, F33, N122, K123, F124	V11, N122, F124

## Data Availability

The datasets used and/or analyzed during the current study are available from the corresponding author upon reasonable request.

## Abbreviations

SGs, stress granules; G3BP1, Ras GTPase-activating protein-binding protein 1; eIF2α, eukaryotic initiation factor 2 alpha; NTF2L, nuclear transport factor 2-like; RRM, RNA recognition motif; Tg, thapsigargin; TC41, top-ranked compound 41; CHX, cycloheximide; ISRIB, Integrated Stress Response Inhibitor; MTT, [4,5-dimethylthiazol-2-yl]-2,5diphenyltrazolium bromide; PFA, Paraformaldehyde; BSA, bovine serum albumin; PBS, phosphate-buffered saline; DAPI, 4′,6-diamidino-2-phenylindole; SDS-PAGE, sodium dodecyl sulfate–polyacrylamide gel electrophoresis; PVDF, polyvinylidene fluoride; DMSO, dimethyl sulfoxide; DMEM, Dulbecco’s Modified Eagle Medium; FBS, Fetal Bovine Serum; A/A, Antibiotic–Antimycotic; RPMI, Roswell Park Memorial Institute; ER, Endoplasmic Reticulum; mTOR, Mammalian Target of Rapamycin; S6K, S6 Kinase; RFP, Red Fluorescent Protein; FGDF, phenylalanine–glycine–aspartic acid–phenylalanine; PDB, Protein Data Bank; (R), Rectus; (S), Sinister.
